# Downregulation of LEMD1-AS1 and Its Influences on the Diagnosis, Prognosis, and Immune Infiltrates of Epithelial Ovarian Cancer

**DOI:** 10.1155/2022/6408879

**Published:** 2022-08-03

**Authors:** Xiaoju Yang, Shen Zhou, Chunlin Li, Li Huang, Chuanqi Chen, Xiao Tang, Xingyan Su, Hongcheng Zhu

**Affiliations:** ^1^Department of Obstetrics and Gynaecology, The Central Hospital of Enshi Tujia and Miao Autonomous Prefecture, Enshi, 445000 Hubei Province, China; ^2^Clinical Laboratory Center, The Central Hospital of Enshi Tujia and Miao Autonomous Prefecture, No. 158 Wuyang Avenue, Enshi City, 445000 Hubei Province, China; ^3^Department of Radiology, The Central Hospital of Enshi Tujia and Miao Autonomous Prefecture, No. 158 Wuyang Avenue, Enshi City, 445000 Hubei Province, China; ^4^Department of Infection, Enshi Tujia and Miao Autonomous Prefecture Center for Disease Control and Prevention, Hubei Province, China; ^5^Department of Anesthesiology, The Central Hospital of Enshi Tujia and Miao Autonomous Prefecture, Enshi City, 445000 Hubei Province, China

## Abstract

Previous studies have confirmed long noncoding RNA LEMD1-AS1 (LEMD1-AS1) as a functional factor in several tumors. The present work is aimed at exploring the prognostic and diagnostic values of LEMD1-AS1 in patients with epithelial ovarian cancer (EOC). We examined the expressions of LEMD1-AS1 in pan-cancer from TCGA microarray datasets and GTEx Project. The expressions of LEMD1-AS1 were detected by qRT-PCR in EOC specimens and normal ovarian specimens from 30 EOC patients. The *χ*^2^ test was applied to compare the clinicopathological characteristics of different groups. ROC curves were established to determine the diagnostic values of LEMD1-AS1 in screening EOC tissues. The association of LEMD1-AS1 expression with clinical outcome was determined by the Kaplan-Meier methods and COX assays. A decreased expression of LEMD1-AS1 was observed in EOC tissues compared to matched normal specimens (*p* < 0.01). Low LEMD1-AS1 expression could be used to distinguish EOC from adjacent normal specimens. A clinical study revealed that patients with low LEMD1-AS1 expression have a shorter overall survival (*p* = 0.035) and progress-free interval (*p* = 0.041) than those with high LEMD1-AS1 expression. The Spearman correlation test revealed that LEMD1-AS1 expressions were negatively associated with the expressions of neutrophil and myeloid dendritic cell. Overall, our finding suggested that LEMD1-AS1 may have potential roles as a potential biomarker and/or a therapeutic target in EOC.

## 1. Introduction

Epithelial ovarian cancer (EOC) is one of the three most common gynecological malignant neoplasms and the third most common cancer in females worldwide [1]. It is anticipated that more than 255,000 women will be diagnosed with EOC each year, which would result in at least 120,000 fatalities per year throughout the world [2]. EOC is characterized by multiple distant metastases in other organisms, advanced-stage appearances, and refractory ascites when firstly diagnosed [3, 4]. Despite various improvements in surgical technology, radiotherapy, or chemotherapy for EOC treatments, the 5-year overall survivals of many EOC patients are still dissatisfied [5, 6]. The major obstacles in the treatments of EOC are the metastasis and multidrug resistance [7]. Therefore, it is necessary to develop a novel and reliable biomarker to evaluate the prognosis and efficacy of therapeutic strategies for EOC.

Numerous studies have demonstrated, as a result of significant developments in high-throughput RNA sequencing technology, that the majority of the human transcriptome may be categorized as long noncoding RNAs (lncRNAs) [8]. lncRNAs are noncoding RNAs over 200 nucleotides in length [9]. Growing studies have confirmed that this class of RNAs shows a regulator effect on the modulation of gene expressions at both transcriptional and posttranscriptional levels [10]. A large number of studies on the basic and clinical assays have revealed that lncRNAs are involved in various biological processes and are distinctly dysregulated in various types of diseases, especially in neoplasms [11, 12]. For instance, lncRNA HAS2-AS1 was significantly expressed in EOC, and it regulated the miRNA-466/RUNX2 axis in a way that made EOC cells more likely to proliferate and metastasize [13]. Upregulation of lncRNA NORAD was shown to promote EOC cell proliferation and decrease bufalin chemoresistance via sponging miR-155-5p [14]. Over the course of the past few years, an increasing number of research have shed light on the possibility that lncRNAs could be utilized as novel biomarkers in the diagnosis of a wide variety of tumor patients. Several biomarker-related lncRNAs have been clinically identified, such as lncRNA SNHG1, lncRNA HOTAIR, and lncRNA MEG3 [15–17]. However, many functional lncRNAs remained unknown.

Long noncoding RNA LEMD1 antisense RNA 1 (LEMD1-AS1) is a recently identified lncRNA. So far, its roles in tumors were rarely reported. In this study, we focused on LEMD1-AS1 and explored its expression and clinical significance in EOC.

## 2. Patients and Methods

### 2.1. Expression Analysis of LEMD1-AS1 and Sample Data across Cancers

The pan-cancer RNA sequencing data relating to 33 different forms of cancer were obtained by downloading them from the Internet database UCEC, which came from TCGA database. In addition, the sequencing information for LEMD1-AS1 was obtained from the GTEx Project. All of the expression data were normalized by converting them to log_2_ format. Evaluation of LEMD1-AS1 expression was performed with the help of the edgeR package in R. 427 EOC samples and 88 normal samples were included for further assays. Clinicopathological features of all patients from TCGA datasets are shown in Table 1.

### 2.2. Patients and Specimens

From 2020 to 2021, 30 EOC patients who underwent complete resection of the tumor in the Central Hospital of Enshi Tujia and Miao Autonomous Prefecture were subsequently enrolled in our study. A total of 30 EOC specimens and nontumor samples were collected from all cases and were flash frozen in liquid nitrogen following surgery. The inclusion criteria are as follows. All patients were diagnosed as EOC by pathological findings; none of the patients had a history of other tumor or received preoperative treatment. Written informed consent was obtained from all cases. Our experiments were approved by the Research Ethics Committee of the Central Hospital of Enshi Tujia and Miao Autonomous Prefecture.

### 2.3. Real-Time PCR

Total RNA was extracted from all specimens with TRIzol reagent (Invitrogen, Xuhui, Shanghai, China). RNA concentration was examined by the use of a spectrometer under a wavelength of 260 nm. cDNA was reverse transcribed using the Quantitect Reverse Transcription Kit (Qiagen, Kunming, Yunnan, China). Then, qRT-PCR was performed to quantify relative LEMD1-AS1 expression using the SYBR Premix Ex Taq kit (TaKaRa, Kunming, Yunnan, China) on CFX99 Real-Time PCR Detection System (Bio-Rad, Hangzhou, Zhejiang, China). GAPDH was used as endogenous control with the 2^−*ΔΔ*Ct^ methods applied for the calculation of the relative expressions of LEMD1-AS1. The sequences of the primers were listed as follows: LEMD1-AS1 forward (5′-3′): AATGACCGCAATCCCAAGGT, LEMD1-AS1 forward (5′-3′): GGTGACTAGCAGTGCGTGAT, GAPDH forward (5′-3′): GGAGCGAGATCCCTCCAAAAT, and GAPDH reverse (5′-3′): GGCTGTTGTCATACTTCTCATGG.

### 2.4. TIMER Database Analysis

TIMER, which can be found at https://cistrome.shinyapps.io/timer/, is a database that was created for the purpose of analyzing immune cell infiltrates in a variety of malignancies. This database used a statistical methodology that has been validated by a pathological investigation in order to estimate the amount of immune infiltration that a tumor has, including neutrophils, macrophages, dendritic cells, B cells, and CD4/CD8 T cells. Using the TIMER database, we first investigated the differences in LEMD1-AS1 expression levels between the various types of tumors. Next, we investigated the association between LEMD1-AS1 expression and the degree of infiltration by the various immune cell subsets. Finally, we drew conclusions about the significance of these findings.

### 2.5. Statistical Analysis

SPSS software (version 16.0; Chicago, IL, USA) and R (version 3.6.0) were applied to perform statistical analyses. Differences between measured groups were assessed using Student's *t*-test or chi-square test. The Kaplan-Meier method was used to calculate survival, and significance was determined by the log-rank test. Receiver-operating characteristic (ROC) curves were used to assess the feasibility of the application of LEMD1-AS1 expression used as a diagnostic tool for detecting EOC. Multivariate analysis was performed to investigate the prognostic factors. *p* < 0.05 was regarded as statistically significant.

## 3. Results

### 3.1. Pan-Cancer Analysis of LEMD1-AS1 Expression

**Figure 1:**
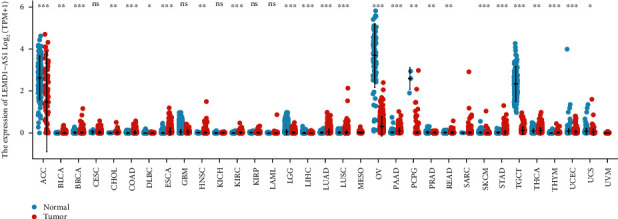
Expression level of LEMD1-AS1 in different cancer types from TCGA and GTEx data. ^∗^*p* < 0.05, ^∗∗^*p* < 0.01, and ^∗∗∗^*p* < 0.001.

Using the LEMD1-AS1 expression data for 33 cancers retrieved from TCGA database and GTEx Project, our group observed that LEMD1-AS1 expression was decreased in several types of tumors, including ACC, BRCA, LGG, LIHC, OV, PCPG, PRAD, SKCM, TGCT, UCEC, and UCS tissues compared to their corresponding normal tissues. However, increased LEMD1-AS1 expression was found in BLCA, CHOL, COAD, DLBC, ESCA, HNSC, KIRC, LUAD, LUSC, PAAD, READ, STAD, THCA, and THYM (Figure 1). Our findings suggested that LEMD1-AS1 may display a different role in tumor progression based on the specific types of tumors.

### 3.2. LEMD1-AS1 Was Lowly Expressed in EOC Tissues

**Figure 2:**
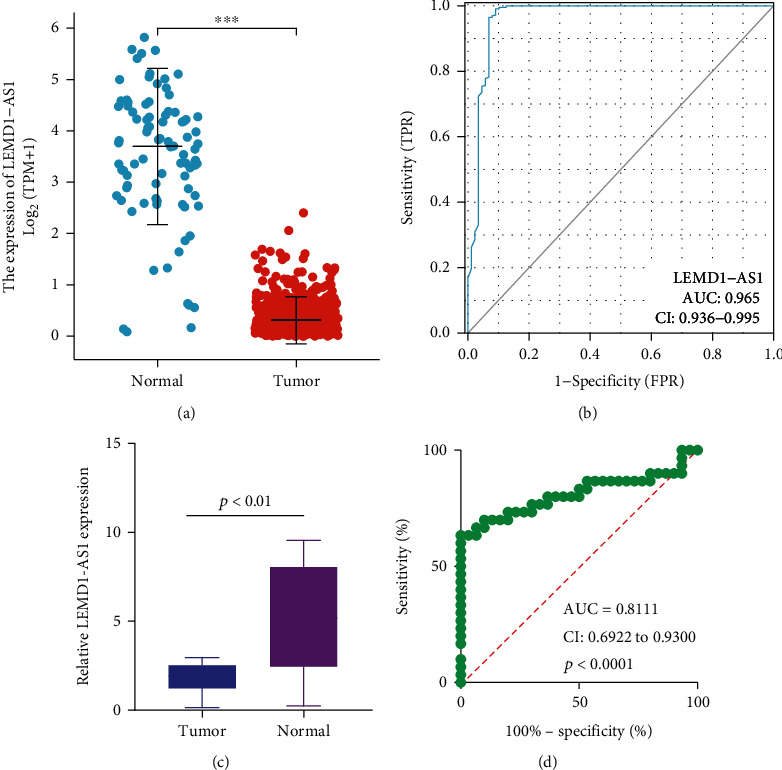
The distinct upregulation of LEMD1-AS1 in EOC patients. (a) The expression of LEMD1-AS1 in EOC specimens and nontumor samples based on TCGA and GTEx data. (b) ROC curve analysis of the diagnostic performance of LEMD1-AS1 expression using TCGA and GTEx data. (c) LEMD1-AS1 was analyzed by RT-PCR assays in 30 EOC tissues and adjacent nontumor specimens from 30 patients. (d) The diagnostic value of LEMD1-AS1 was demonstrated in our cohort. ^∗∗∗^*p* < 0.001.

Based on the data of TCGA database and GTEx Project, we found that LEMD1-AS1 expression was distinctly decreased in EOC samples compared with normal samples (Figure 2(a)). The diagnostic value of lncRNAs in EOC patients had been frequently reported in several studies. Thus, we further explored the diagnostic value of LEMD1-AS1. As shown in Figure 2(b), the results of ROC assays suggested that LEMD1-AS1 effectively differentiated EOC specimens from normal specimens with an area under the ROC curves (AUC) of 0.965 (95% CI: 0.936 to 0.995). Moreover, we further determined whether LEMD1-AS1 expression was dysregulated in EOC in our cohort. As shown in Figure 2(c), RT-PCR results showed that LEMD1-AS1 expressions were distinctly decreased in EOC samples compared with the noncancerous specimens (*p* < 0.01), implying that deregulated expression of LEMD1-AS1 could play a role in the developments of EOC. Moreover, the results of ROC assays suggested that LEMD1-AS1 effectively differentiated EOC specimens from normal specimens with an area under the ROC curves (AUC) of 0.8111 (95% CI: 0.6922 to 0.9300) (Figure 2(d)).

### 3.3. Association of LEMD1-AS1 Expressions with Clinicopathological Features of EOC

We split all 379 EOC patients into a high expression group (*n* = 190) and a low expression group (*n* = 189) based on the mean expression of LEMD1-AS1 in the 379 EOC specimens. This was done so that we could gain a better knowledge of the clinical significance of LEMD1-AS1 expressions in EOC. Then, the chi-square test was performed for the statistics assays. As presented in Table 1, there were no distinct connections between the dysregulated expressions of LEMD1-AS1 and any other clinical characteristics (all *p* > 0.05).

### 3.4. High Levels of LEMD1-AS1 Were Correlated with Unfavorable Survivals in EOC

**Figure 3:**
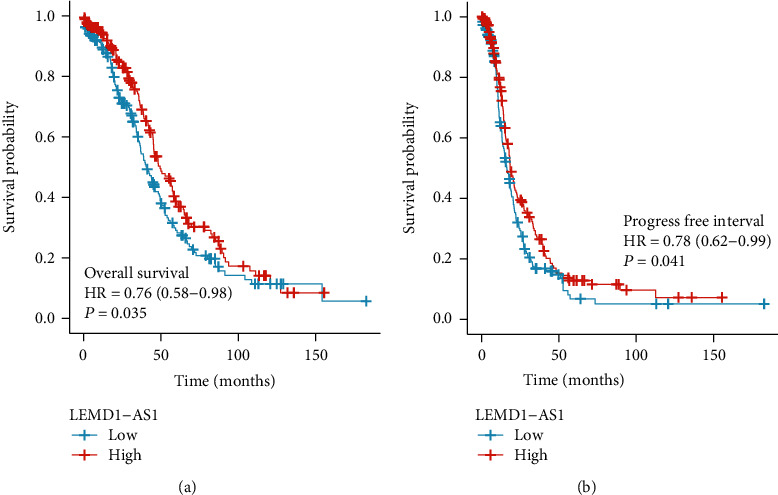
Kaplan-Meier curves estimating the (a) overall survival and (b) progression-free interval rates according to the expression of LEMD1-AS1 in patients with EOC.

To study whether the abnormal expression of LEMD1-AS1 influences the clinical outcome of EOC patients, we performed Kaplan-Meier analysis for the statistical assays, finding that patients with low LEMD1-AS1 expression have a shorter overall survival (*p* = 0.035, Figure 3(a)) and progress-free interval (*p* = 0.041, Figure 3(b)) than those with high LEMD1-AS1 expression. For further exploration of the prognostic value of LEMD1-AS1 levels in EOC patients, we performed univariate analysis which revealed that primary therapy outcome, stage, age, and LEMD1-AS1 expression were related to the clinical outcome of EOC patients (Table 2). However, in multivariate analysis, we just observed that age and primary therapy outcome were an independent prognostic factor for overall survival of EOC patients (Table 2).

### 3.5. The Association between LEMD1-AS1 Expression and Immune Infiltration

**Figure 4:**
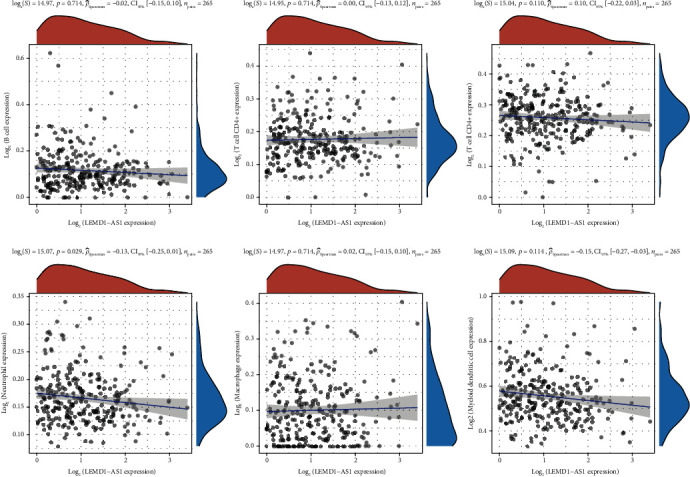
Correlation of LEMD1-AS1 expression with immune infiltration level in EOC.

The Spearman correlation test was applied to explore the relationships between LEMD1-AS1 expressions and immune cell infiltration by the use of TIMER. We observed that LEMD1-AS1 expression was negatively associated with the levels of neutrophil and myeloid dendritic cell (Figure 4).

## 4. Discussion

EOC is the eighth most common cause of death from cancer in women. The clinical prognosis of EOC patients remained poor after the comprehensive treatments, including surgery, chemotherapy, and radiotherapy [18, 19]. The early diagnosis contributed to a favorable long-term survival of EOC patients, and the early prediction of clinical outcome could guide the targeted therapies and optimize therapeutic schedules [20]. However, up to date, the sensitive and specific biomarkers are limited in clinical practice. In recent years, an increasing number of studies have suggested that lncRNAs have the potential to be employed as novel biomarkers due to the specific dysregulation of lncRNAs and their capacity to behave as tumor promoters or oncogenes [21, 22].

Recently, more and more studies have demonstrated that the expression abnormalities of lncRNAs were associated with the progression of many cancers. For instance, Wang et al. firstly indicated that lncRNA B3GALT5-AS1 expression was distinctly decreased in colon cancer and promoted suppressed colon cancer liver metastasis via the miRNA-203/EMT axis, suggesting that LEMD1-AS1 served as an oncogenic lncRNA in this tumor [23]. Qian et al. reported that lncRNA MIR4435-2HG expressions were distinctly increased in lung cancer and predicted an advanced stage and distant metastasis. Functional experiments revealed that MIR4435-2HG silence suppressed the proliferation and metastasis of lung cancer cells via by activating *β*-catenin signalling. To date, only a study by Guo et al. reported that there was a downregulation of LEMD1-AS1 expression in both the OC tissues and the OC cell lines. In addition, the regulation of miR-183-5p and TP53 by forced upregulation of LEMD1-AS1 decreased the proliferation and metastasis of ovarian cancer cells. However, the expression and function of LEMD1-AS1 in other tumors have not been investigated.

In this research, LEMD1-AS1 was demonstrated to be lowly expressed in EOC specimens based on TCGA datasets and our cohort. Then, ROC assays revealed LEMD1-AS1 as a useful tool to distinguish EOC specimens from normal ovarian tissues. A clinical study indicated that patients with low LEMD1-AS1 expression have a shorter OS and PFI than those with high LEMD1-AS1 expression. We performed univariate analysis which revealed that primary therapy outcome, stage, age, and LEMD1-AS1 expression were associated with the clinical outcome of EOC patients. However, in multivariate analysis, we just observed that age and primary therapy outcome were an independent prognostic factor for overall survival of EOC patients. In addition, we offered evidences that LEMD1-AS1 may be exploited as an innovative diagnostic and prognostic biomarker for EOC patients.

There is a correlation between immune infiltration of tumor cells and the spread of EOC to lymph nodes, as well as the patient's prognosis [24, 25]. An examination of the TIMER database revealed that the expression of LEMD1-AS1 was inversely linked with the expression of both neutrophil and myeloid dendritic cell. It is essential for antitumor immunity for T cells to get activated and expand while they are in the tumor microenvironment [26]. As a result of complement-dependent T cell suppression, neutrophils in the TME become unique from myeloid-derived suppressor cells in their ability to block T cell proliferation and activity. Based on these findings, it appeared that LEMD1-AS1 may play a role in the immunological response of EOC tumors to their surrounding microenvironment.

## 5. Conclusions

We identified LEMD1-AS1 as a novel EOC-related lncRNA which was lowly expressed in EOC specimens and predicted a poor prognosis. We found the correlation of LEMD1-AS1 with neutrophil and myeloid dendritic cell. Overall, we confirmed that LEMD1-AS1 could be applied as a potential biomarker of EOC diagnosis and prognosis. However, the specific function and exact molecular mechanisms of LEMD1-AS1 in EOC remain unclear and require further investigation.

## Figures and Tables

**Table 1:** Association between LEMD1-AS1 expression and clinicopathological characteristics of EOC.

Characteristic	Low expression of LEMD1-AS1	High expression of LEMD1-AS1	*p*
*n*	189	190	
FIGO stage, *n* (%)			0.151
Stage I	0 (0%)	1 (0.3%)	
Stage II	7 (1.9%)	16 (4.3%)	
Stage III	152 (40.4%)	143 (38%)	
Stage IV	29 (7.7%)	28 (7.4%)	
Primary therapy outcome, *n* (%)			0.109
PD	18 (5.8%)	9 (2.9%)	
SD	11 (3.6%)	11 (3.6%)	
PR	26 (8.4%)	17 (5.5%)	
CR	100 (32.5%)	116 (37.7%)	
Race, *n* (%)			0.501
Asian	4 (1.1%)	8 (2.2%)	
Black or African American	13 (3.6%)	12 (3.3%)	
White	165 (45.2%)	163 (44.7%)	
Age, *n* (%)			0.646
≤60	101 (26.6%)	107 (28.2%)	
>60	88 (23.2%)	83 (21.9%)	
Histologic grade, *n* (%)			0.874
G1	0 (0%)	1 (0.3%)	
G2	23 (6.2%)	22 (6%)	
G3	163 (44.2%)	159 (43.1%)	
G4	0 (0%)	1 (0.3%)	
Age, median (IQR)	60 (52, 70)	58 (50.25, 66.75)	0.161

**Table 2:** Univariate and multivariate analyses of prognostic factors in EOC patients.

Characteristics	Total (*N*)	Univariate analysis		Multivariate analysis	
		Hazard ratio (95% CI)	*p* value	Hazard ratio (95% CI)	*p* value
FIGO stage	374				
Stage I & stage II	24	Reference			
Stage III	293	2.045 (0.905-4.621)	0.085	2.255 (0.815-6.241)	0.118
Stage IV	57	2.495 (1.057-5.889)	**0.037**	2.441 (0.839-7.100)	0.101
Primary therapy outcome	307				
PD	27	Reference			
SD	22	0.441 (0.217-0.895)	**0.023**	0.443 (0.213-0.918)	**0.029**
PR	42	0.652 (0.384-1.107)	0.113	0.598 (0.341-1.049)	0.073
CR	216	0.152 (0.093-0.247)	**<0.001**	0.150 (0.090-0.251)	**<0.001**
Race	364				
Asian & Black or African American	37	Reference			
White	327	0.637 (0.405-1.004)	0.052	0.711 (0.419-1.206)	0.206
LEMD1-AS1	377				
Low	188	Reference			
High	189	0.757 (0.585-0.981)	**0.035**	0.866 (0.640-1.170)	0.348
Age	377				
≤60	206	Reference			
>60	171	1.355 (1.046-1.754)	**0.021**	1.421 (1.049-1.925)	**0.023**

## Data Availability

The datasets used and/or analyzed during the current study are available from the corresponding author upon reasonable request.
